# Factors associated with hospitalization for seasonal influenza in a Japanese nonelderly cohort

**DOI:** 10.1186/s12889-016-3602-z

**Published:** 2016-09-02

**Authors:** Sachiko Ono, Yosuke Ono, Hiroki Matsui, Hideo Yasunaga

**Affiliations:** 1 Department of Clinical Epidemiology and Health Economics, School of Public Health, The University of Tokyo, 7-3-1 Hongo, Bunkyo-ku, Tokyo, 1130033 Japan; 2 Department of General Medicine, National Defense Medical College, 3-2 Namiki, Tokorozawa, Saitama 3598513 Japan

**Keywords:** Seasonal influenza, Risk factor, Hospitalization, Nonelderly patient

## Abstract

**Background:**

Nonelderly patients may require hospitalization if their symptoms of influenza are severe. However, little evidence is available about the risk factors for hospitalization for influenza.

**Methods:**

We used a multicenter outpatient and inpatient database to obtain patients’ characteristics and clinical procedures. We identified patients aged <65 years with a confirmed diagnosis of influenza between October 2013 and December 2014. We used a Cox regression model to identify the risk factors for hospitalization, using a comparison group of individuals with a confirmed diagnosis of influenza but who were not hospitalized.

**Results:**

Of 88,054 patients diagnosed with influenza, 276 (0.3 %) patients were hospitalized. With reference to patients aged 18–64 years, the hazard ratio (95 % confidence interval) for hospitalization in patients aged <2, 2–4, and 5–17 years was 12.25 (8.37–17.93), 4.56 (3.10–6.72) and 1.45 (1.03–2.05), respectively. Anemia, chronic obstructive pulmonary disease, neurologic disease, and regular steroid use were significantly associated with hospitalization. Hazard ratios were adjusted for age, sex, comorbidities, respiratory co-infection, virus type, and influenza season.

**Conclusions:**

Our findings suggest that younger age and several comorbidities are associated with higher probability of hospitalization for influenza.

## Background

Seasonal influenza is a vaccine-preventable disease that imposes a substantial burden on the global population every year. The disease is estimated to affect 5–10 % of adults and 20–30 % of children, and causes severe illness in 3 million to 5 million patients worldwide each year [1]. Owing to the rarity of fatal cases [2], especially in nonelderly patients, hospitalization for influenza has been the main focus of several studies [3] to make decisions about resource planning, such as stockpiling of medicines, and to decrease the impact of influenza on public health. For example, a previous study estimated the number of hospitalizations for influenza in the United States from national hospital discharge surveillance data, and found that 57 % of all patients requiring hospitalization for influenza were aged <65 years [3]. Another study estimated that 3.4 % of all critical illness hospitalizations were attributable to influenza [4, 5].

Data have been lacking on the risk factors for hospitalization, although individuals at high risk for complicated influenza have been defined [6]. A systematic review in 2013 revealed that the risk factors for hospitalization for influenza were rarely investigated [6]. A recent study reported that age, underlying illness, and influenza viral load were strong predictors for hospitalization in 424 patients with laboratory-confirmed influenza who visited emergency departments [7]. Another study demonstrated that individuals with pulmonary disease, dementia, renal disease, or cancer had a higher likelihood of hospitalization among community-dwelling elderly people [8].

Hospitalization, which may reflect both the severity of disease and physicians’ tendency to hospitalize patients, is considered a measurable outcome for influenza in patients aged <65 years [2]. However, owing to the lack of data, few studies have focused on hospitalization for influenza in nonelderly patients in all clinical settings, including primary care, emergency department, and hospital admissions. The characteristics of the patients may be different in these clinical settings, and the risk factors for hospitalization for influenza in the general population remain unknown.

The aims of the present study were (i) to examine patient demographic characteristics, clinical courses, and complications, and (ii) to identify risk factors for hospitalization in patients aged <65 years diagnosed with influenza, using a multicenter outpatient and inpatient database in Japan.

## Methods

### Data source

For this study, we used the Japan Medical Data Center (JMDC) database. The JMDC database includes annual health checkup records and health insurance claims data for approximately 1.5 million insured individuals in 2013. The majority of the insured individuals are employees of Japanese companies and their families. The database includes information about administrative claims data for clinic visits and hospital admissions. Diagnoses and drugs are recorded based on International Classification of Diseases 10th revision (ICD-10) codes and World Health Organization Anatomical Therapeutic Chemical (WHO-ATC) codes, respectively. The data did not include individual vaccination status. We referred to a national surveillance report to obtain information on seasonal trends and circulating influenza subtypes [9].

Given the anonymous nature of the data, the requirement for informed consent was waived. The study was approved by the Institutional Review Board of The University of Tokyo.

### Case identification and clinical courses

We identified patients aged <65 years diagnosed with influenza (ICD-10 code: J10) and confirmed by influenza testing from October 2013 to December 2014. We excluded data for patients already hospitalized for diseases other than influenza. We examined several comorbidities based on outpatient information recorded within 6 months before the diagnosis of influenza, and selected these comorbidities by reference to a previous publication [6]. Patients with asthma, diabetes mellitus, and steroid use were identified by prescription information using WHO-ATC classification system codes R03 for asthma, A10 for diabetes mellitus, and H02 for steroid use. We coded patients who were prescribed oral steroids and took these steroids for >30 days during the baseline period as those regularly using steroids. ICD-10 codes were used to identify patients with chronic obstructive pulmonary disease (COPD) (J41–J44), cardiovascular disease (I20–I25, Q20–Q28), cerebrovascular disease (I60–I69), mental disorder including dementia (F00–F99), neurologic disease (G00–G99), anemia (D50–D59, D60–D64), immunodeficiency (D80–D89), liver disease (K70–K77), malignancy (D00–D09, C00–C97), and respiratory co-infection (J0, J12-J18 and J2). Influenza encephalitis and influenza pneumonia were identified as complications by Japanese text codes. Japanese medical procedure codes were used to identify the following medical procedures: dialysis; influenza diagnosis test; oxygen administration; peripheral oxygen saturation monitoring; cardiorespiratory monitoring; intubation; ventilation; and admission to intensive care unit. Saturation monitoring and cardiorespiratory monitoring are only recorded when patients are continuously monitored. We defined patients with fatal outcomes as those who died within 1 month of the diagnosis of influenza. We extracted records for prescription of all available neuraminidase inhibitors in Japan: oseltamivir; laninamivir; zanamivir; and peramivir. Although it was not possible to prove causation of neuraminidase inhibitor-induced adverse drug reactions, we identified entries of the following ICD-10 codes that coincided with the prescription of a neuraminidase inhibitor: T375 for adverse effects of antiviral drug; K70–K77 with Japanese text including “drug” for drug-induced hepatic dysfunction; L511 for Stevens–Johnson syndrome (SJS); and T782 for anaphylaxis. We defined October 2013–September 2014 as the 2013/2014 influenza season and October 2014–December 2014 as the 2014/2015 influenza season.

### Data analysis

Continuous data are presented as the mean (± standard deviation, SD), and categorical data are presented as numbers (proportion). The chi-square test for categorical variables and *t*-test for continuous variables were used to compare two groups. We performed a multivariable Cox regression analysis to evaluate factors associated with hospital admission. The comparison group was individuals diagnosed with influenza but who were not hospitalized. For patients who had multiple hospitalizations for influenza during the study period, we only included the first episode in the analysis. Patients in whom the subtype of influenza was not recorded were excluded from the analysis. Variables with P <0.10 in univariate analyses at baseline were included as independent variables in the multivariable analysis. Proportional hazard assumption was confirmed using a graphical technique. We conducted a subgroup analysis restricting of individuals diagnosed with influenza in the 2013/2014 season (from October 2013 to September 2014) to examine possible effect modification between years. All statistical analyses were two-tailed and values of P <0.05 were considered significant. All statistical computations were performed with SPSS version 22 (IBM SPSS, Armonk, NY).

## Results

A total 1,469,320 individuals were observed during the baseline period, and 153,711 left the cohort in the following study period. We identified 88,071 patients aged <65 years diagnosed with influenza and confirmed by influenza testing. More than 95 % of patients received rapid influenza diagnosis tests (RIDTs). We subsequently excluded 17 patients hospitalized before diagnosis of influenza. We analyzed the remaining 88,054 patients, of whom 276 (0.3 %) patients were hospitalized. During the study period, four patients had multiple episodes of hospitalization for influenza. Mean (SD) follow-up period was 14.0 months (3.0 months) for the entire cohort and 14.7 months (1.6 months) for patients diagnosed with influenza. The seasonal peaks and proportions of virus types in the study population were consistent with national surveillance data, which observed A(H1N1), B(Victoria), and B(Yamagata) in the 2013/2014 season, and A(H3) in the 2014/2015 season.

Table 1 shows the clinical characteristics of the eligible patients. Age 18 to 64 years was most common in the hospitalized patients, as well as all comorbidities. Among the hospitalized patients, there were six (2.2 %) with influenza encephalitis and six (2.2 %) with influenza pneumonia. Although the database did not include causes of death, one patient died within 1 month of diagnosis after influenza.

**Table 1:** Clinical characteristics of patients diagnosed with influenza

	Total					2013/2014 season					2014/2015 season				
Parameters	Non-hospitalized patient		Hospitalized patient		*P*	Non-hospitalized patient		Hospitalized patient		*P*	Non-hospitalized patient		Hospitalized patient		*P*
	No.	(%)	No.	(%)		No.	(%)	No.	(%)		No.	(%)	No.	(%)	
Number of patients	87,778		276			74,101		239			13,677		37		
Female, *n* (%)	39,122	(44.57)	141	(51.09)	0.034	32,896	(44.39)	122	(51.05)	0.045	6226	(45.52)	19	(51.35)	0.585
Age, (years)					<0.001		0.00			<0.001					
<2	2916	(3.32)	58	(21.01)		2553	(3.45)	56	(23.43)		363	(2.65)	2	(5.41)	
2–4	7932	(9.04)	58	(21.01)		6934	(9.36)	48	(20.08)		998	(7.30)	10	(27.03)	
5–17	35,066	(39.95)	75	(27.17)		29,397	(39.67)	62	(25.94)		5669	(41.45)	13	(35.14)	
18–64	41,864	(47.69)	85	(30.80)		35,217	(47.53)	73	(30.54)		6647	(48.60)	12	(32.43)	
Virus type					<0.001		0.00			<0.001					0.769
A	54,643	(62.25)	188	(68.12)		41,160	(55.55)	152	(63.60)		13,483	(98.58)	36	(97.30)	
B	32,048	(36.51)	78	(28.26)		31,863	(43.00)	77	(32.22)		185	(1.35)	1	(2.70)	
Both A and B	1030	(1.17)	10	(3.62)		1021	(1.38)	10	(4.18)		9	(0.07)	0	(0.00)	
Unknown	57	(0.06)	0	(0.00)		57	(0.08)	0	(0.00)		0	(0.00)	0	(0.00)	
Comorbidity															
Asthma	20,631	(23.50)	109	(39.49)	<0.001	17,536	(23.66)	99	(41.42)	<0.001	3095	(22.63)	10	(27.03)	0.659
COPD	444	(0.51)	8	(2.90)	<0.001	382	(0.52)	7	(2.93)	<0.001	62	(0.45)	1	(2.70)	0.422
Cardiovascular disease	811	(0.92)	5	(1.81)	0.222	705	(0.95)	5	(2.09)	0.14	106	(0.78)	0	0.00	1.000
Cerebrovascular disease	357	(0.41)	4	(1.45)	0.025	304	(0.41)	3	(1.26)	0.126	53	(0.39)	1	(2.70)	0.352
Diabetes mellitus	811	(0.92)	6	(2.17)	0.065	711	(0.96)	4	(1.67)	0.425	100	(0.73)	2	(5.41)	0.019
Mental disorder	4294	(4.89)	25	(9.06)	0.002	3618	(4.88)	22	(9.21)	0.003	676	(4.94)	3	(8.11)	0.612
Neurologic disease	4425	(5.04)	33	(11.96)	<0.001	3708	(5.00)	26	(10.88)	<0.001	717	(5.24)	7	(18.92)	0.001
Anemia	1749	(1.99)	15	(5.43)	<0.001	1458	(1.97)	12	(5.02)	0.002	291	(2.13)	3	(8.11)	0.052
Immunodeficiency	113	(0.13)	1	(0.36)	0.811	97	(0.13)	1	(0.42)	0.741	16	(0.12)	0	0.00	1.000
Liver disease	1768	(2.01)	11	(3.99)	0.035	1494	(2.02)	6	(2.51)	0.755	274	(2.00)	5	(13.51)	<0.001
Dialysis	19	(0.02)	1	(0.36)	0.08	14	(0.02)	1	(0.42)	0.039	5	(0.04)	0	0.00	1.000
Malignancy	547	(0.62)	6	(2.17)	0.004	464	(0.63)	4	(1.67)	0.102	83	(0.61)	2	(5.41)	0.008
Regular steroid use	796	(0.91)	8	(2.90)	0.002	665	(0.90)	7	(2.93)	0.003	131	(0.96)	1	(2.70)	0.808
Complication															
Respiratory co-infection	59,597	(67.90)	201	(72.83)	0.092	50,589	(68.27)	177	(74.06)	0.064	9008	(65.86)	24	(64.86)	1.000
Encephalitis	0	(0.00)	6	(2.17)	<0.001	0	(0.00)	6	(2.51)	<0.001	0	(0.00)	0	(0.00)	–
Pneumonia	0	(0.00)	6	(2.17)	<0.001	0	(0.00)	4	(1.67)	<0.001	0	(0.00)	2	(5.41)	<0.001
Death	0	(0.00)	1	(0.36)	<0.001	0	(0.00)	1	(0.42)	<0.001	0	(0.00)	0	(0.00)	–

*COPD* chronic obstructive pulmonary disease

Table 2 shows the clinical practice patterns for influenza in outpatients and hospitalized patients. Some hospitalized patients were administered a neuraminidase inhibitor before admission. Neuraminidase inhibitors were prescribed for >95 % of outpatients and >90 % of hospitalized patients. Approximately 64 % of hospitalized patients did not receive continuous saturation/cardiorespiratory monitoring or respiratory support in the initial month of hospitalization; these parameters would have been used intermittently.

**Table 2:** Clinical practice patterns for influenza

Practice	Non-hospitalized patient		Hospitalized patient	
	No.	%	No.	%
Number of patients	87,778		276	
Neuraminidase inhibitor^a^				
Oseltamivir	29,729	(33.87)	70	(25.36)
Laninamivir	35,014	(39.89)	27	(9.78)
Zanamivir	14,788	(16.85)	15	(5.43)
Peramivir	2563	(2.92)	87	(31.52)
Combinations	2149	(2.45)	58	(21.01)
None	3535	(4.03)	19	(6.88)
Procedures				
Oxygen administration			43	(15.58)
Saturation monitoring			71	(25.72)
Cardiorespiratory monitoring			59	(21.38)
ICU admission			1	(0.36)
None of above			177	(64.13)

^a^For hospitalized patients, neuraminidase inhibitors prescribed before admission are included

**Fig. 1:**
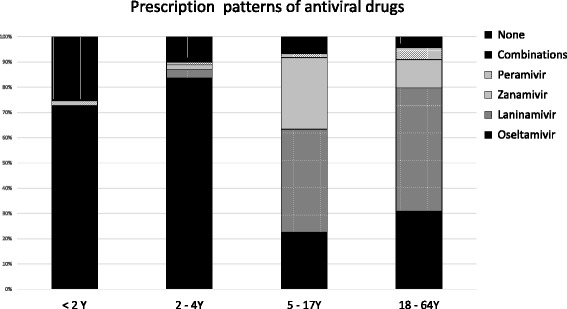
Prescription patterns of neuraminidase inhibitors

Figure 1 shows the prescription of neuraminidase inhibitors in each age category. The highest proportion of peramivir administration was observed in patients aged 18–64 years, while oseltamivir was predominantly used in patients aged <4 years (72.7–83.6 %); the proportion of oseltamivir was lowest in patients aged 5–17 years (22.6 %). Although there were no recorded adverse effects of antiviral drugs, one case of SJS, four cases of drug-induced hepatic dysfunction, and nine cases of anaphylaxis coincided with the prescription of neuraminidase inhibitors.

Table 3 shows the results of the multivariable Cox regression analysis for hospitalization. Patients aged <2 years (hazard ratio (HR), 12.25; 95 % confidence interval (CI), 8.37–17.93; P <0.001), 2–4 years (HR, 4.56; 95 % CI, 3.10–6.72; *P* < 0.001), and 5–17 years (HR, 1.45; 95 % CI, 1.03–2.05; *P* = 0.003) were at higher risk of hospitalization. Comorbidities that had significant associations with hospitalization were anemia, COPD, neurologic disease, and regular steroid use. In the subgroup analysis restricted to patients diagnosed with influenza in the 2013/2014 season, age <5 years, COPD, neurologic disease, and regular steroid use were significantly associated with hospitalization (Table 4).

**Table 3:** Results of Cox regression analysis for hospitalization in patients diagnosed with influenza

	Univariate Analysis			Multivariable analysis		
	HR	95.0 % CI	*P*	HR	95.0 % CI	*P*
Female	1.30	1.03 to 1.65	0.029	1.26	0.99 to 1.59	0.059
Age, (years)						
18–64	Ref.		<0.001	Ref.		<0.001
<2	9.69	6.94 to 13.53	<0.001	12.25	8.37 to 17.93	<0.001
2–4	3.58	2.56 to 5.00	<0.001	4.56	3.10 to 6.72	<0.001
5–17	1.05	0.77 to 1.43	0.757	1.45	1.03 to 2.05	0.033
Comorbidity						
Asthma	2.12	1.66 to 2.70	<0.001	1.15	0.88 to 1.51	0.309
COPD	5.79	2.87 to 11.70	<0.001	3.71	1.75 to 7.88	0.001
Cerebrovascular disease	3.59	1.34 to 9.64	0.011	2.48	0.88 to 6.97	0.084
Diabetes mellitus	2.37	1.05 to 5.32	0.037	2.18	0.92 to 5.19	0.076
Mental disorder	1.93	1.28 to 2.92	0.002	1.41	0.89 to 2.25	0.144
Neurologic disease	2.56	1.78 to 3.68	<0.001	2.62	1.68 to 4.11	<0.001
Anemia	2.82	1.67 to 4.74	<0.001	1.90	1.09 to 3.31	0.024
Liver disease	2.02	1.10 to 3.69	0.023	1.47	0.76 to 2.83	0.255
Dialysis	16.09	2.26 to 114.66	0.006	5.87	0.77 to 44.89	0.088
Malignancy	3.53	1.57 to 7.93	0.002	2.29	0.96 to 5.43	0.061
Regular steroid use	3.22	1.60 to 6.51	0.001	2.35	1.12 to 4.91	0.024
Respiratory co-infection	1.26	0.97 to 1.65	0.082	1.06	0.81 to 1.38	0.689
Virus type						
A	Ref.		<0.001	Ref.		0.002
B	0.71	0.54 to 0.92	0.009	0.73	0.55 to 0.96	0.025
Both A and B	2.80	1.48 to 5.28	0.002	2.21	1.16 to 4.22	0.016
Season						
2013/2014	Ref.			Ref.		
2014/2015	0.83	0.59 to 1.17	0.283	0.82	0.57 to 1.17	0.267

*COPD* chronic obstructive pulmonary disease

**Table 4:** Results of Cox regression analysis for hospitalization in patients diagnosed with influenza in the 2013/2014 season

	Univariate Analysis			Multivariable analysis		
	HR	95.0 % CI	*P*	HR	95.0 % CI	*P*
Female	1.31	1.01 to 1.68	0.039	1.26	0.98 to 1.63	0.075
Age, (years)						
18–64	Ref.		<0.001	Ref.		<0.001
<2	10.46	7.38 to 14.81	<0.001	12.02	8.07 to 17.91	<0.001
2–4	3.32	2.31 to 4.79	<0.001	3.82	2.52 to 5.82	<0.001
5–17	1.02	0.72 to 1.43	0.924	1.31	0.90 to 1.91	0.155
Comorbidity						
Asthma	2.27	1.76 to 2.94	<0.001	1.24	0.93 to 1.66	0.139
COPD	5.72	2.70 to 12.14	<0.001	4.09	1.84 to 9.09	0.001
Cerebrovascular disease	3.08	0.99 to 9.62	0.053	2.28	0.70 to 7.47	0.173
Diabetes mellitus	1.74	0.65 to 4.69	0.270	1.77	0.63 to 4.99	0.282
Mental disorder	1.97	1.27 to 3.06	0.002	1.58	0.96 to 2.59	0.074
Neurologic disease	2.32	1.54 to 3.48	<0.001	2.39	1.45 to 3.94	0.001
Anemia	2.62	1.47 to 4.68	0.001	1.77	0.95 to 3.29	0.072
Liver disease	1.25	0.56 to 2.82	0.586	0.90	0.38 to 2.15	0.816
Dialysis	20.76	2.91 to 147.95	0.002	9.34	1.18 to 74.03	0.034
Malignancy	2.70	1.00 to 7.24	0.049	2.00	0.71 to 5.65	0.192
Regular steroid use	3.29	1.55 to 6.98	0.002	2.58	1.18 to 5.64	0.018
Respiratory co-infection	1.32	0.99 to 1.77	0.057	1.10	0.82 to 1.48	0.509
Virus type						
A	Ref.		<0.001	Ref.		0.002
B	0.65	0.49 to 0.86	0.002	0.73	0.55 to 0.97	0.028
Both A and B	2.62	1.38 to 4.97	0.003	2.23	1.17 to 4.26	0.015

*COPD* chronic obstructive pulmonary disease

## Discussion

To the best of our knowledge, this is the largest study to have investigated the clinical courses and risk factors for hospitalization in patients diagnosed with influenza in all clinical settings. The proportion of hospitalized patients was 0.3 % in 88,054 patients with test-confirmed influenza. More than 90 % of patients diagnosed with influenza were prescribed neuraminidase inhibitors. Patients aged <17 years were at higher risk of hospitalization than those aged 18–64 years. Comorbidities identified as risk factors for hospitalization included anemia, COPD, neurologic disease, and regular steroid use.

To date, there is no conclusive evidence of the age at which risk of hospitalization for influenza is highest. We observed the strongest association between influenza hospitalization and age <2 years compared with other child age categories. Interestingly, a previous meta-analysis reported that children aged <2 years were at lower risk of hospitalization than children aged 2–17 years, although the investigators judged the grade of the evidence to be very low due to study heterogeneity and lack of adjustment for confounding factors [6]. The findings of a more recent meta-analysis focusing on children chime with our adjusted hazard ratios, identifying the strongest association between hospitalization and age in those aged <2 years compared with children in other age categories [10]. We also found that the risk of hospitalization after diagnosis of influenza was elevated in children 2–5 years and 5–17 years, albeit to a lesser extent than those aged <2 years. Currently, the WHO and Centers of Disease Control and Prevention recommend that children aged 6 months to 5 years be vaccinated against influenza. Considering the potential harm and restricted use of antivirals for patients aged 5–17 years in current practice [11], inclusion of this age category in vaccination recommendations may help avoid hospital admission for influenza.

Although a previous meta-analysis reported any chronic lung disease as a risk factor for hospitalization without discriminating between asthma and COPD [6], we observed that only COPD was a significant risk for hospitalization. In addition to the different categorization of pulmonary diseases, this inconsistency may be partially attributable to another methodological differences: the assessment in the previous study was unadjusted, whereas ours was adjusted for multiple factors. We also found that anemia, COPD, neurologic disease, and regular steroid use were significant risk factors for hospitalization for influenza. These findings are broadly comparable with those of previous studies [1, 12]. Further targeting of individuals with these risk factors for influenza vaccination may help minimize their suffering, the economic impact caused by hospitalization, and potential exacerbation of the underlying medical condition.

We observed that >90 % of patients diagnosed with influenza were prescribed neuraminidase inhibitors. A recent meta-analysis showed that oseltamivir alleviated the symptoms of influenza in children [13]. In the present study, the proportion of oseltamivir use was lowest in patients aged 5–17 years. In current practice in Japan, oseltamivir is not recommended for children aged >10 years because of a report in 2005 describing an association with neuropsychiatric adverse effects in adolescents [14], despite the fact that the relationship between those events and oseltamivir was unclear and a conclusive judgment about causation could not be made [15]. Oseltamivir is currently the only neuraminidase inhibitor that can be administered orally to patients with underlying respiratory disease, because laninamivir and zanamivir are administered as inhaled powders and have the potential to reduce the forced expiratory volume in 1 second [16]. In addition, peramivir is not recommended for use in outpatients [14]. Because there is little available evidence on the effects of oseltamivir on hospital admission in children [13], further conclusive studies will be needed to examine the clinical benefits and potential harm of oseltamivir for children aged ≥10 years.

Our study had several limitations. First, the data did not include day of symptom onset, severity of influenza, pregnancy, socioeconomic status, or vaccination status. Owing to the lack of information on vaccination status, the results potentially underestimated the impacts of well-known risk factors, including severe cardiovascular disease, renal failure, pulmonary disease including asthma, and extreme age, because individuals with these risk factors were likely to have been vaccinated. Currently, the Japanese government only subsidizes influenza vaccination for individuals aged ≥65 years and those aged ≥60 years with chronic disease. Vaccination coverage in other populations is not fully understood, but was reportedly 59.2 % in children aged <13 and 28.6 % in individuals aged 13–64 years in the 2010–11 season [17]. Another study that investigated 2935 Japanese individuals with asthma reported that 63.6 % were vaccinated in the 2011–12 influenza season [18]. Thus, no significant association between hospitalization and other well-known risk factors, including asthma may have been affected by influenza vaccination. On the other hand, despite high vaccination coverage among children, it is notable that age <17 years was still identified as a risk for hospitalization in our study. Second, the results may have been affected by the exclusion of patients with false-negative results in RIDTs because the test was reported to have a specificity of approximately 60 % [19]. Third, the generalizability of our findings may be limited because accessibility to medical care may be higher in Japan than other countries, because of Japan’s universal healthcare system. Also, our study population does not completely reflect that of Japan as a whole, as our cohort comprised employees of Japanese companies with health insurance and their families; the cohort did not include unemployed adults. In addition, A (H1N1), A (H3), B (Victoria), and B (Yamagata) were the most frequently observed subtypes of influenza virus during the study period, and the results may not be able to be extrapolated to other strains of influenza virus. In fact, our results indicated possible effect modification between influenza seasons likely influenced by virus strain, as the significant association between hospitalization and ages 5–17 years was absent in the subgroup analysis that examined only data from the 2013/2014 season. Finally, because comorbidities recorded in an administrative claims database are generally less accurate than those in planned prospective studies, potential miscoding of comorbidities may have affected the results if there was an imbalance of miscoding between hospitalized patients and non-hospitalized patients.

Nevertheless, the strength of our study is that we identified risk factors for hospitalization using a database that covers a very large number of individuals diagnosed with influenza in all clinical settings.

## Conclusion

In conclusion, we found that people aged <17 years were at higher risk for hospitalization for influenza than people aged 18–64 years. Comorbidities as risk factors for hospitalization were anemia, COPD, neurologic disease, and regular steroid use. People with these conditions should be further encouraged to be vaccinated against influenza as a preventative measure against hospitalization.

## Abbreviations

COPD: chronic obstructive pulmonary disease
ICD-10: International Classification of Diseases 10th revision
JMDC: Japan Medical Data Center
RIDTs: Rapid influenza diagnosis tests
SJS: Stevens–Johnson syndrome
WHO: World Health Organization
WHO-ATC: World Health Organization Anatomical Therapeutic Chemical
